# Studies of Antibiotic Resistance of Beta-Lactamase Bacteria under Different Nutrition Limitations at the Single-Cell Level

**DOI:** 10.1371/journal.pone.0127115

**Published:** 2015-05-20

**Authors:** Ying Wang, Min Ran, Jun Wang, Qi Ouyang, Chunxiong Luo

**Affiliations:** 1 The State Key Laboratory for Artificial Microstructures and Mesoscopic Physics, School of Physics, Peking University, Beijing, China; 2 Center for Quantitative Biology, Academy for Advanced Interdisciplinary Studies, Peking University, Beijing, China; 3 Peking-Tsinghua Center for Life Sciences, Peking University, Beijing, China; University of Birmingham, UNITED KINGDOM

## Abstract

Drug resistance involves many biological processes, including cell growth, cell communication, and cell cooperation. In the last few decades, bacterial drug resistance studies have made substantial progress. However, a major limitation of the traditional resistance study still exists: most of the studies have concentrated on the average behavior of enormous amounts of cells rather than surveying single cells with different phenotypes or genotypes. Here, we report our study of beta-lactamase bacterial drug resistance in a well-designed microfluidic device, which allows us to conduct more controllable experiments, such as controlling the nutrient concentration, switching the culture media, performing parallel experiments, observing single cells, and acquiring time-lapse images. By using GFP as a beta-lactamase indicator and acquiring time-lapse images at the single-cell level, we observed correlations between the bacterial heterogeneous phenotypes and their behavior in different culture media. The feedback loop between the growth rate and the beta-lactamase production suggests that the beta-lactamase bacteria are more resistant in a rich medium than in a relatively poor medium. In the poorest medium, the proportion of dormant cells may increase, which causes a lower death rate in the same generation. Our work may contribute to assaying the antibiotic resistance of pathogenic bacteria in heterogeneous complex media.

## Introduction

Antibiotics are able to cure diseases because they can efficiently inhibit cell wall synthesis, protein synthesis, or DNA replication to kill pathogenic bacteria or inhibit their growth.[1] However, bacteria have become resistant to many antibiotics as a result of gene mutations or the exchange of genetic segments via plasmids or transposons.[2] The abuse of antibiotics in the community, in agriculture, and in hospitals has exacerbated the crisis of antibiotic resistance.[3,4] Worse, many resistant bacteria not only survive antibiotic therapy themselves but also protect other sensitive bacteria from antibiotics through secreting hydrolases for antibiotics into their common environment.[5,6]

Bacterial tolerance comes from many sources, including antibiotic hydrolytic enzymes[7,8], dormant bacteria[9–11], and others. All of these sources of tolerance can be classified into two types: genotype and phenotype tolerance.[12] The mechanism of phenotype tolerance is the functional heterogeneity of the bacteria: a subpopulation of bacteria shows a zero growth rate, so the bacterial population is persistent to antibiotics via an “insurance policy”. In other words, the population always contains a small fraction of dormant cells, which can mutually transform into normal cells spontaneously.[13,14] The mechanism of genotype tolerance is that a cell possess an improved resistant ability compared with a wild-type cell through gene mutation or exchange.[2]

Previous studies have shown that sensitive bacteria with lower growth rates have higher survival rates.[12] Efforts have been made to study the drug persistence of sensitive bacteria at different growth rates, in which a chemostat was often used to analyze the number of CFUs (colony-forming units) or the MIC (minimal inhibitory concentration) of bacteria, which cannot distinguish the behavior of single cells.[15] Such studies may give misleading information when the bacteria change their phenotypes.

Recently, studies have reported that the expression of drug-resistance genes and related proteins may be controlled by the growth rate, which in turn influences the survival rate under antibiotic stress.[16–18] Recent advances in microfluidics facilitate the study of single-cell dynamic behavior and the control of the cellular microenvironment.[19–22] These methods enable us to study the sensitive and drug-resistant strains under different antibiotic concentrations with single-cell resolution. However, it is still interesting to know how the resistant strains respond to antibiotics under different nutrition conditions with single-cell resolution because different nutrient concentrations will change the bacterial growth rate. That effect is the focus of this study.

**Fig 1:**
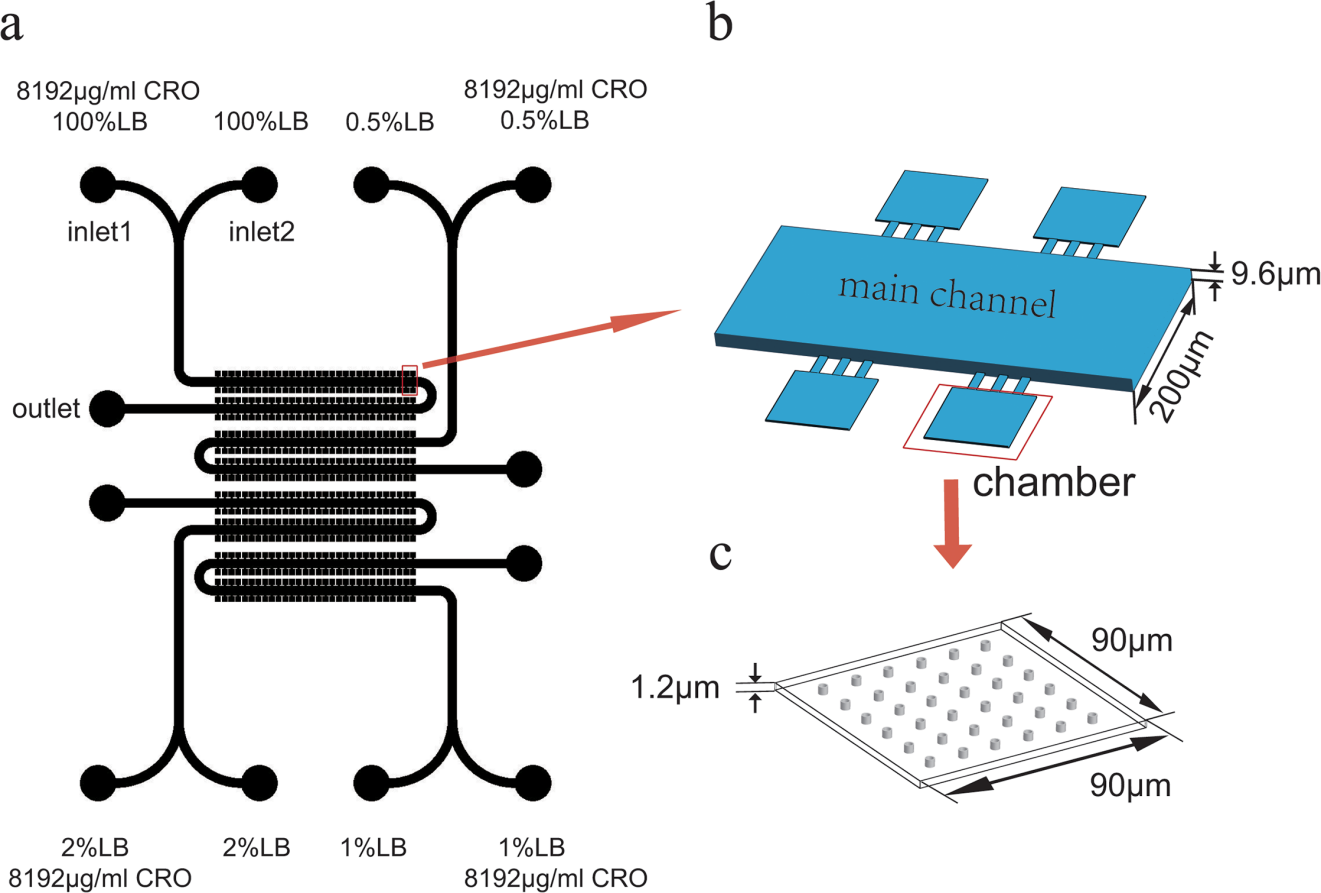
Experimental setup in our study. (a) top view of the whole chip design. There are 4 parallel identical units with observing area concentrated together; each unit comprises 2 inlets, 1 outlet, a main channel and 120 chambers. The experimental medium in syringes connected to inlets is shown. CRO is an abbreviation for Ceftriaxone Sodium. (b) 3D view of a select area of Fig 1A. The main channel is 9.6 μm high, 200 μm wide, and 20 mm long. Three rectangles connect the chamber and the main channel; this part is 10 μm wide, 40 μm long, and 1.2 μm high. (c) 3D view of a chamber. The chamber’s three-dimensional size is 90μm*90μm*1.2μm. Because the chamber’s area-to-height ratio is too large, we designed 36 pillars to prevent the chamber from collapsing. The pillar diameter is 3 μm. The distance between every pillar is 10 μm.

Flow cytometry has been used generally in the single-cell data acquiring.[23–26] However, flow cytometry provides only instant information (such as cell size, protein level) of single cell and cannot track a single cell of interest.[27] Thus, it can hardly distinguish the heterogeneous dynamic behavior of bacteria when facing stress. In this paper, we used a microfluidic chip with many 1.2 μm high micro-chambers (Fig 1), which allow monitoring bacterial behavior at the single-cell level in a constant-medium environment in parallel.[28] It is convenient to observe phenotype changes (such as cell length, growth rate, fluorescence intensity and cell death with high time resolution) in our microfluidic device. Those single-cell information about phenotype variation are essential in giving more information and explanations for antibiotic therapy. A beta-lactam ceftriaxone-resistant *Escherichia coli* (strain DH5α) was studied in this chip with different concentrations of nutrients and unique concentrations of antibiotics. This drug-resistant strain contains plasmids bearing a CTX gene fragment, which can express beta-lactamase to hydrolyze beta-lactams. Meanwhile, the plasmids transcribe GFP as a beta-lactamase indicator. With time-lapse image acquisition at the single-cell level, we observed correlations between the heterogeneous phenotypes of *Escherichia coli* and their behavior in different nutrition condition.[29] We found that the beta-lactamase resistant *E*. *coli* with lower growth rates had higher GFP intensity, which indicates a higher beta-lactamase concentration under the same conditions. In addition, when adding antibiotics, we found that the bacterial growth rate decreased, and the GFP intensity increased. The beta-lactamase bacteria were even more resistant in rich medium (100% LB) than in relatively poor medium (2% LB). Furthermore, in the poorest medium (0.5% LB), the population of dormant cells may increase, which causes a lower death rate in the same generation. Our study about the persistent patterns of drug-resistant cells in different nutrition conditions may inspire further investigations concerning drug resistance in heterogeneous environments.

## Materials and Methods

### Microfluidic device design and fabrication

As Fig 1 shows, the chip contains four repeated units, which are convenient for multiple sets of repetitive experiments. Each unit has two inlets for switching the culture media, one outlet, and one main channel. There are 120 chambers connected to both sides of each main channel. The chamber is 1.2 μm high, slightly larger than the diameter of *E*. *coli*. This dimension can align the bacteria to form a single layer in the chambers, which is convenient for observing and tracing each single cell. The mold of the chip was fabricated using standard 2-layer lithography method. [30,31] After replicating the channel pattern from the mold to PDMS, we bonded the PDMS with glass after oxygen plasma treatment.[32] Before cell loading, the chip was sterilized by UV disinfection for 30 minutes.

### Strain and culture

The strain we used in the experiment was derived from *E*. *coli* (strain DH5α). This resistant strain was transformed with PUA66 plasmids, which contain a gene segment co-expressing the CTX beta-lactamase enzyme and green fluorescent protein (see S1 Fig for more details)[33]. The antibiotic we used in this study was Ceftriaxone Sodium (CRO for short. Bought from Rocephine Company), a type of β-lactam antibiotic. This resistant DH5α strain began to die under 8192 μg/ml CRO[28]. A single clone was inoculated into 4 ml of LB in the previous day, which was supplemented with 2 μg/ml CRO to maintain the plasmids. After culturing overnight at 220 rpm at 37°C, 200 ml bacterial solution was diluted by a factor of 20 using fresh LB supplemented with 2 μg/ml CRO, then continued to culture for 1.5 hours to activate the bacteria. Finally, we initiated the cell loading.

### Operation and observation

First, we centrifuged the activated bacterial solution and re-suspended it with four different culture media (100% LB, 2% LB, 1% LB, 0.5% LB, diluted with PBS, all supplemented with 2 μg/ml CRO) for 1 to 2 hours, then we suctioned off the re-suspension solution with four syringes. Because the height of the chamber (1.2 μm) is close to the diameter of *E*. *coli*, it was difficult for the cells to enter into the chamber. To solve this problem, we first blocked the two inlets with a polyethylene pipe, and then connected the syringe to the outlet with another polyethylene pipe and push the cell suspension into the outlet. By doing this, we created an extruding force which facilitate the cell to enter into the chamber effectively. Approximately 100 μl of cell solution was injected into each unit, as a result, every chamber contained approximately 5–10 cells.

After that, we replaced the syringe at the outlet with another syringe loaded with corresponding concentration of culture medium and pressed the syringe to generate a high flow velocity to wash away the cells in the main channel.

Next, we fixed the chip in the observation platform of microscope. Two inlets of each unit were connected to two syringes; one of them contained 1 ml of a different concentration of LB solution (2 μg/ml CRO), and the other one contained 8192 μg/ml CRO in the corresponding LB solution. These syringes were distributed as shown in Fig 1A. Every outlet was connected to a tube to collect the waste. Each syringe was manipulated by a programmable automatic control pump. The flow velocity was set at 50 μl/hour, which made the medium conditions in the chamber almost equivalent to those in the main channel (see S1 File for more details). We programmed the pump to inject LB (containing 2 μg/ml CRO) during the first hour, and then switched to inject LB (containing 8192 μg/ml CRO) during the next 8 hours. We used a Nikon Ti microscope, which was equipped with a perfect focus system and an automatic motorized stage, to pick tens of chambers of interest in each unit and take pictures of each chamber every 5 minutes. Note the time scale that we were interested in is approximately 6 hours. In this short time scale, any genetic mutation of the bacteria during the experiments could be safely ruled out, so we could focus on studying the bacterial response strategy to different drug stresses.

## Results

### Comparison of Bacterial phenotype change in different nutrition concentration

In order to monitor the *E*. *coli* and facilitate analysis of the bacterial growth rate and death rate, we marked the DH5α strain with green fluorescent protein, which is co-expressed with the hydrolytic enzyme. Thus, the expression level of the hydrolytic enzyme can be represented by the fluorescence intensity of the GFP. As was found earlier, in Jiang’s work[28], the drug-resistant bacteria began to die when the CRO concentration was increased to 8192 μg/ml. At 1 hour of the experiment, we put the bacteria in a lethal antibiotic concentration (8192 μg/ml), took fluorescent images every 5 minutes with the microscope. Then we analyzed the bacterial growth rate and enzyme expression level by measuring the bacterial length and fluorescence intensity, respectively.

**Fig 2:**
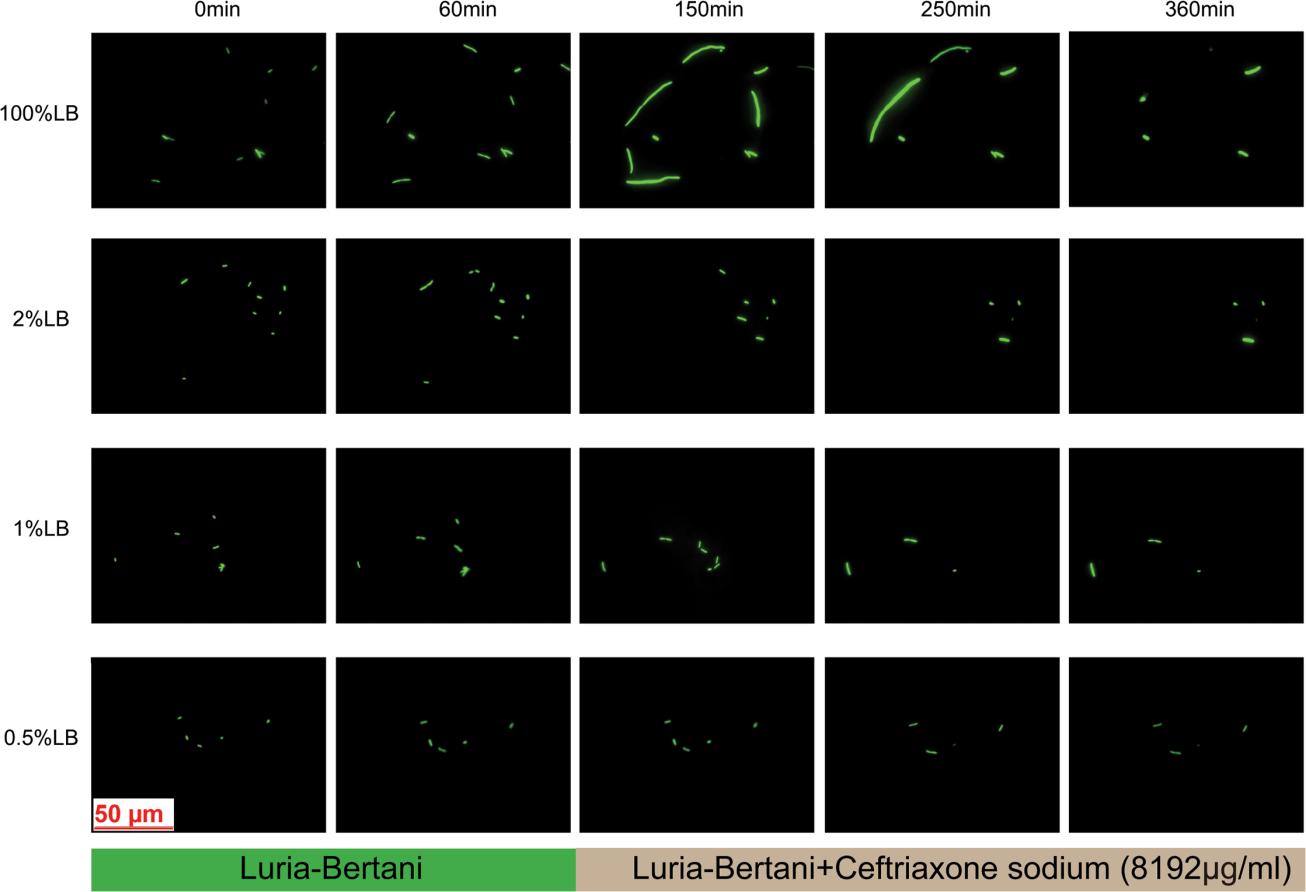
Representative time sequence images in different nutrition conditions. Under different diluted nutrition conditions, the time sequence changes the bacterial phenotypes in the chamber. The syringes only injected LB solution (supplemented with 2 μg/ml CRO) into the chip in the first hour, then the syringe was switched to a different LB solution, containing 8192 μg/ml CRO. The fluorescence intensity of the bacteria make sense when relatively comparing in the same condition.

Examples of temporal change are shown in Fig 2. The bacteria were cultured in medium (supplemented with 2 μg/ml CRO) during the first hour in order to let the bacteria to adapt to the current culture conditions. Over this period, we clearly found that the bacteria elongate and divide in 100% LB, while almost no growth in 0.5% LB.

Next, we switched to the nutrition supplemented with 8192 μg/ml CRO at a time point of 1 hour. As Fig 2 indicates, in 100% LB, most of the bacteria continued elongating and intensifying fluorescence after we added the antibiotics. At 150 or 250 minutes, some of the bacteria began to bubble or quenched their fluorescence and finally died, but the bacteria with low growth rates survived. In comparison, under the condition of diluted nutrients, the ratio of elongating bacteria was very small; and the growth rate was also low, especially in 0.5% LB medium, where the bacteria hardly change their phenotype. We note that in 100% LB, the fluorescence intensity of the bacteria kept increasing after adding antibiotics, while it decreased gradually in 0.5% LB.

### Statistical results of bacterial growth rate and fluorescence intensity

**Fig 3:**
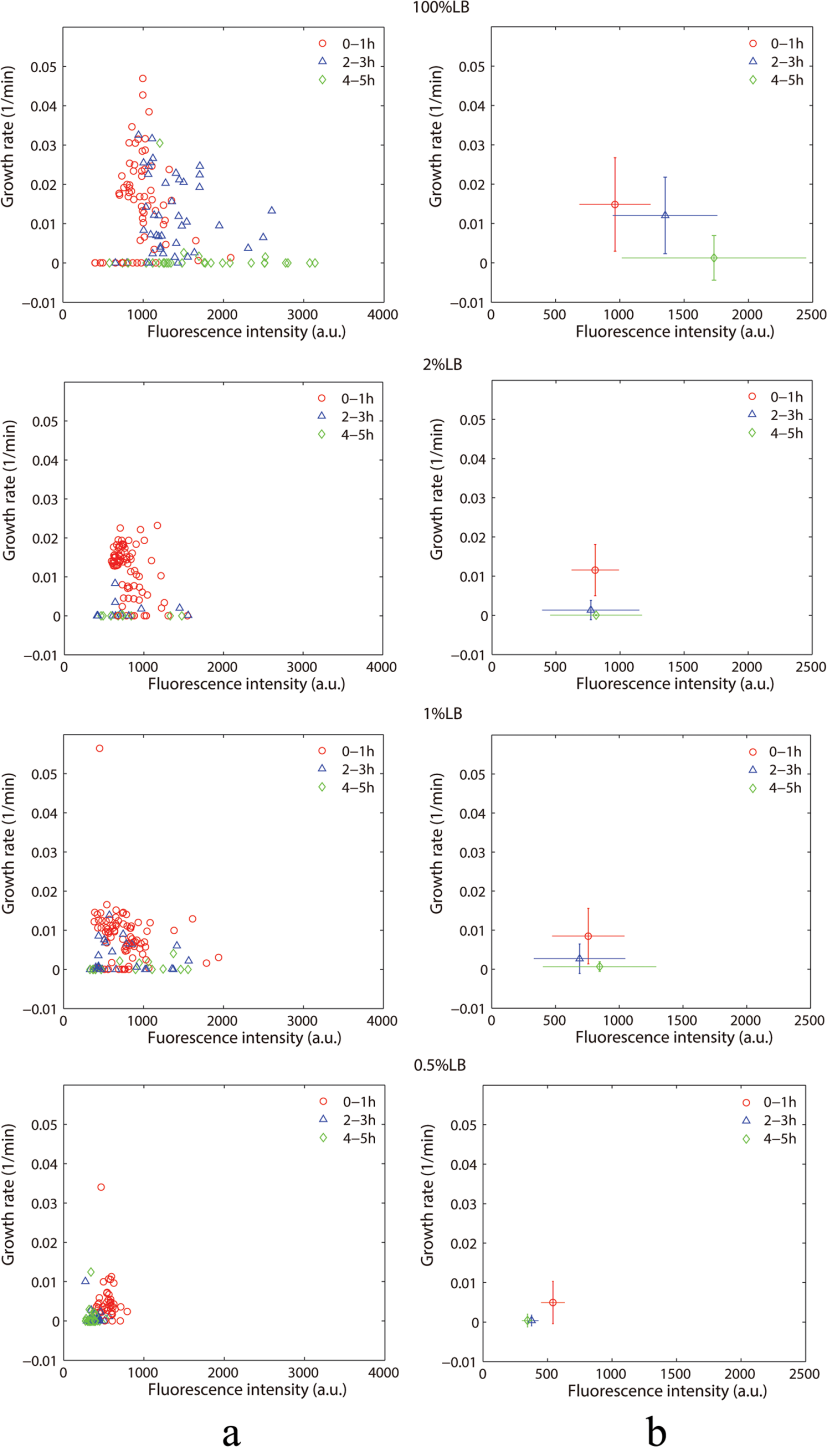
Statistical scatter diagram of the growth rate and the fluorescence intensity. (a) A scatter diagram of the bacterial growth rate and the fluorescence intensity. Each points represents a cell. The vertical axis represents the average individual bacterial growth rate within a one-hour time period; the horizontal axis represents the average fluorescence intensity of single bacteria within a 1-hour time period. Different colors represent different time periods. Bacterial growth rates less than zero are set to zero. (b) The average bacterial growth rate and the fluorescence intensity in different time periods. The error bars in the vertical and horizontal directions represent the standard deviation of the growth rate and the fluorescence intensity, respectively.

Using the multi-chamber chip shown in Fig 1, we conducted a series of experiments under different nutritional conditions. An image processing program developed with Matlab was employed to analyze the fluorescent images. The length of the bacteria (L) was measured, and the growth rate (k) of the bacteria (defined as k = dL/dt)/L) was calculated for each bacterium. Because antibiotics can lead to changes in the bacterial growth rate, we chose the images in the first hour for determining the initial growth rate k0 (we have confirmed that the bacterial growth rate remains unchanged in the first hour, See S2 Fig for more details). In addition, we also tracked and recorded the division and death of each cell and its fluorescence intensity. The relationship between the growth rate and the fluorescence intensity is shown in Fig 3.

Fig 3A shows a scatter plot of the bacterial growth rate and the fluorescence intensity over different time periods. It can be seen that the distribution of the bacterial growth rate in 100% LB tends to be more extensive, ranging from 0 to 0.05. The growth rate mean value is approximately 0.015; that is, the generation time of the bacteria is approximately 65 minutes. With the dilution of nutrients, the distribution of the bacterial growth rate was more concentrated in a range from 0 to 0.01. The average growth rate of the bacteria became smaller.

In the meantime, the bacterial fluorescence intensity in 100% LB increased substantially from the first stage (0–1 h) to the second stage (2–3 h); the increase ratio (**Ir** = I_2–3_/I_0–1_) of fluorescence intensity is approximately 1.5. The fluorescence intensity increase while the growth rate decrease as time lapse, that is, the GFP intensity has an approximately negative relationship with the bacterial growth rate (Fig 3B 100%LB). Because the fluorescence intensity represents the expression quantity of antibiotic hydrolases, this result means that the cells chose to secrete a large number of hydrolases to degrade antibiotics in response to the pressure from antibiotics. (The growth rate and GFP fluorescence intensity remained constant in the first hour; see S2 Fig)

As a comparison, the increase ratio **Ir** decreased under the low-nutrition concentration. Under 2%, 1%, and 0.5% LB, the fluorescence intensity decreased from the first stage (0–1 h) to the second stage (2–3 h); that is, **Ir**<1. This result indicates that the expression of hydrolytic enzymes decreases while the growth rate decreases under the pressure of antibiotics in the low nutrition conditions, which are quite different with 100% LB. We also find that death rates of bacteria in 1% and 2% LB are much higher than that of 100% LB condition. However, death rate of bacteria is very low in the case of 0.5% LB. That means the reason of persistence in 0.5% LB is not the expression of hydrolytic enzymes. This result occurs because the cell growth rate is almost zero under 0.5% LB condition(and the cells are more inclined to turn into the dormant state); the death rate under 0.5% LB was much lower.

### Survival rate of bacteria in different nutrition concentrations

**Fig 4:**
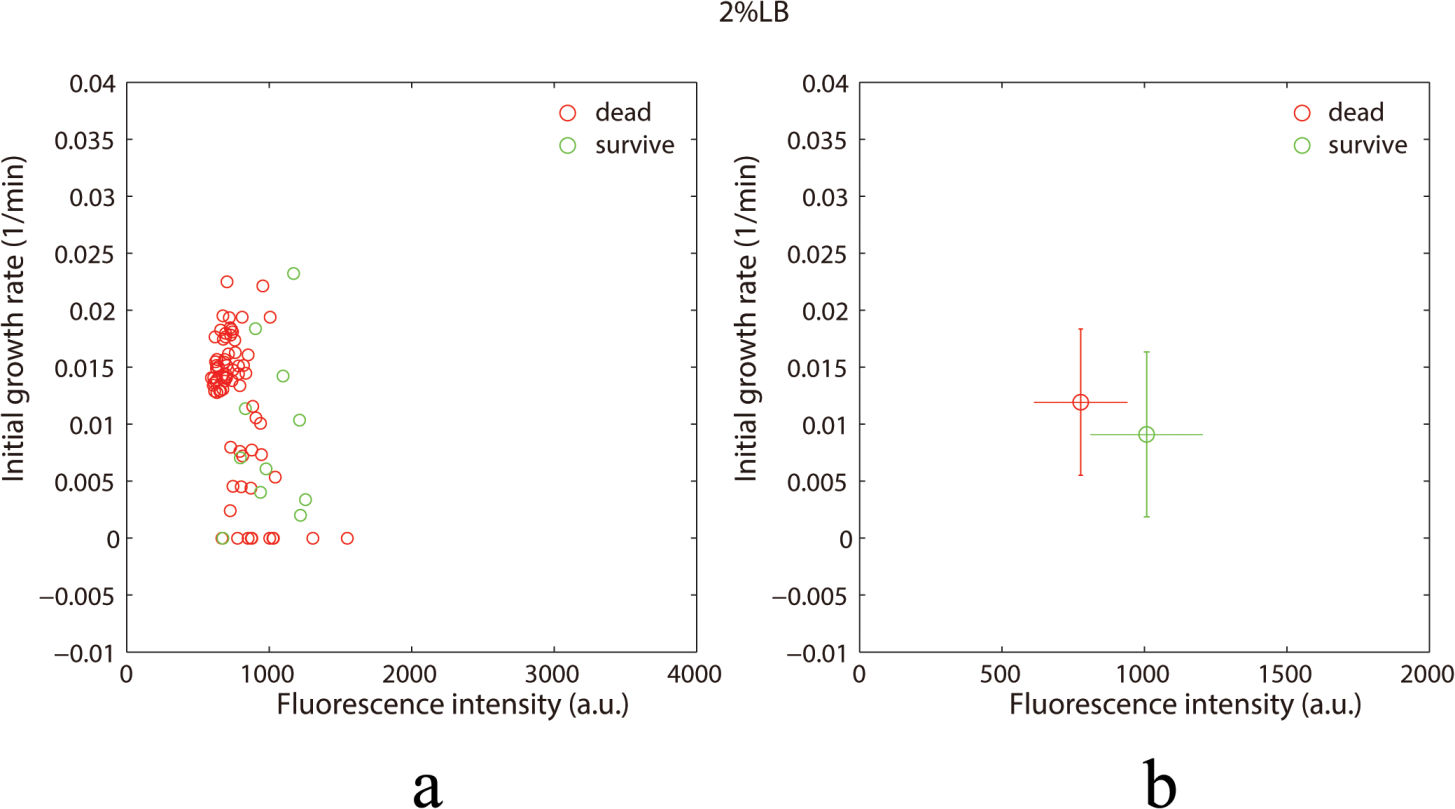
Relationship between cell death and initial growth rate. (a) the scatter diagram for the initial growth rate and the fluorescence intensity in the first hour. Red and green represent the cells that were dead and survived at the time point of 7 hours. (b) the average initial growth rate and fluorescence intensity. The error bars in the vertical and horizontal directions represent the standard deviation of the growth rate and the fluorescence intensity, respectively.

We tracked the trajectory of each bacterium and observed whether it died or not (If the cell bubbled or quenched its fluorescence and eventually disappeared, then we regarded this cell was died). At the same time, we recorded the initial growth rate and the fluorescence intensity of the dead and surviving cells, respectively. As indicated in Fig 4, K_0_ refers to the growth rate in the first hour. Red represents bacteria that eventually died, and green represents bacteria that survived 6 hours after adding antibiotic. We found a negative relationship between the growth rate and the hydrolytic enzyme concentration of the bacteria under the same nutrition conditions (for example, 2% LB). Most of the bacteria that eventually died had larger initial growth rates and lower fluorescence intensities. The initial heterogeneous states of bacteria cause different bacterial fates eventually.

**Fig 5:**
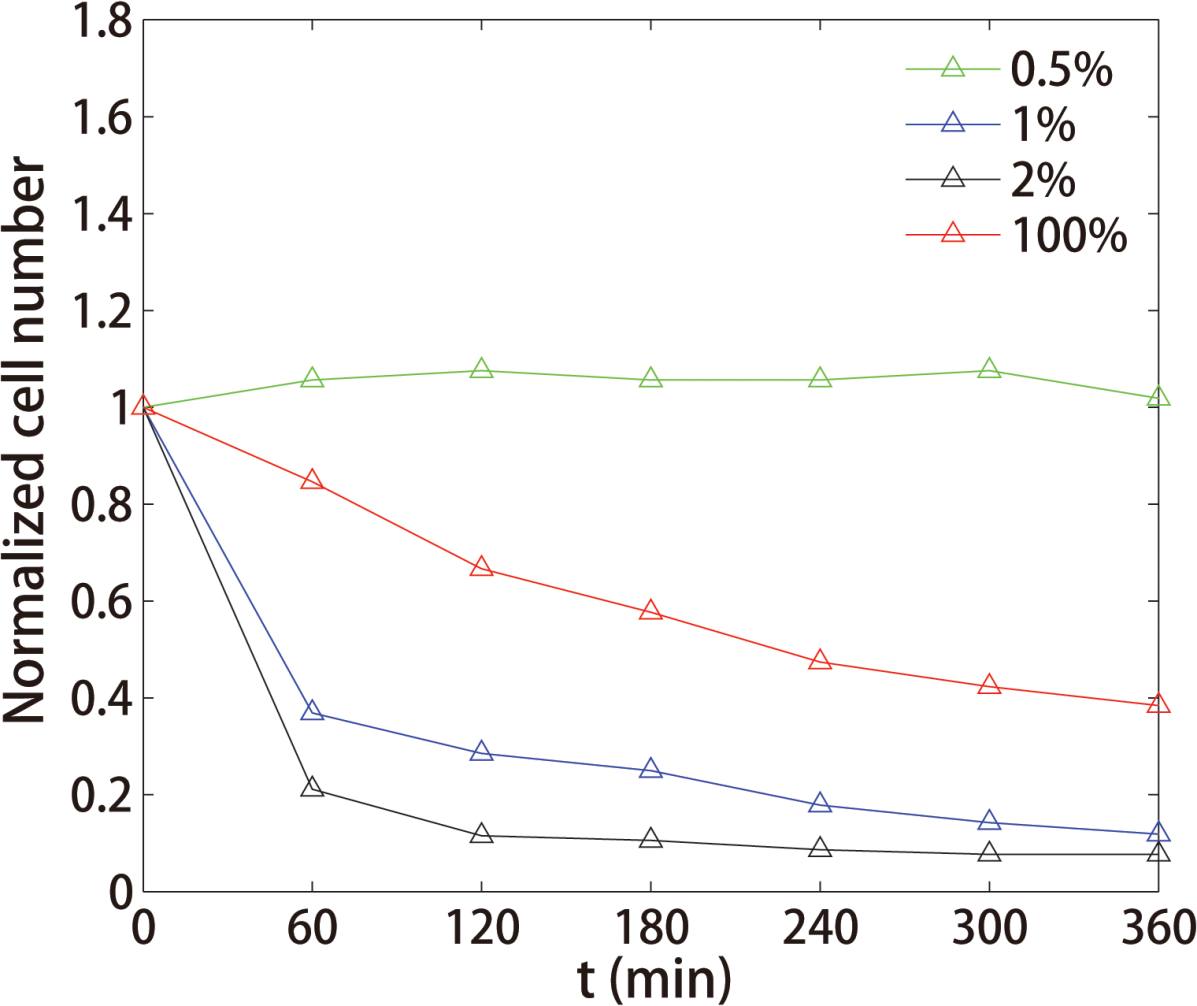
Survival rate in different nutrition conditions. The normalized number of cells changed from 0 minutes (adding 8192 μg/ml CRO at this time) to 360 minutes in different nutrient dilutions. The vertical axis shows the survival rate. The relative rank of the survival rate at 6 hours after adding CRO was 2% <1% <100% <0.5%.

In Fig 5, we counted the total cell number every 60 minutes in every nutrition condition to calculate the survival rate of the bacterial population. We found that the survival rates in the cases of 0.5% and 100% LB were higher than those of 1% and 2% LB (Fig 5). In the case of 100% LB, the bacterial survival rate is enhanced because of the higher concentration of beta-lactamase to degrade CRO compared with the 2% or 1% LB cases. Thus, as the experiments show, a limited but not extremely poor nutrition condition (2% LB, for example) is more effective for killing drug-resistant bacteria.

## Discussion

In this paper, we utilized our microfluidic system and the image processing program to distinguish the dynamics of bacterial resistant behaviors at single cell level in different nutrition conditions and found some innovational results as follows (All the data underlying these results are within S2 File):

In the case of 100% LB, when adding antibiotics (8192 μg/ml), the bacteria not only greatly increased the expression level of the enzyme to degrade the antibiotics but also began to decrease the growth rate. However, the death rate was still high for those bacteria with large initial growth rates. With the decrease of nutrition concentration from 100% to 2% or 1%, the decrease of bacterial growth rate caused little increase of beta-lactamase concentration as shown in Fig 3. Meanwhile, the bacterial death rate increased. These results suggested that under relatively low nutrition level, breakdown of feedback relation [28,33] between growth rate and beta-lactamase level may increase the death rate of resistant cell.

When the nutrient concentration was reduced to 0.5%, the death rate is lower than in the case of 2% and 1%, even lower than in the undiluted LB. From single cell data, we found that the bacteria have very low growth rate and low beta-lactamase level under 0.5% LB. It could be explained as follows: with the deterioration of the nutritional conditions, the bacteria chose another mechanism: the cells reduced their protein expression level and bacterial growth rate when confronted with antibiotic stress. When more bacteria have slower growth rate or even turn into dormant state in the poorest nutrition condition, the bacteria will have low cell wall synthesizing rate, hence the health of cell wall is not substantially affected by the CRO. As a result, it improves the resistant capacity considerably. In another words, under the effect of antibiotics, bacteria take the nutrition conditions into account and choose to increase the expression level of antibiotic hydrolases or reduce their protein expression level and bacterial growth rate as a response to antibiotic pressure. For the first time, we observed the transformation of resistant mechanism along with the dilution of nutrition concentration at the single-cell level, which can provide a new insight not only for researching the single cell antibiotic resistance, but also for analysis of population resistance in heterogeneous complex media. Our research also provides an enlightenment to the clinical therapy for pathogenic bacteria. In some heterogeneous nutrition conditions, the traditional approach of using a high concentration of antibiotics to kill drug-resistant bacteria cannot be guaranteed to be effective.

## Supporting Information

S1 Fig Construction of the plasmid expressing CTX-M-14. (PDF)

S2 Fig Growth rate and fluorescence intensity remain constant in the first hour. (PDF)

S1 File Supporting information of the manuscript. (DOC)

S2 File All data underlying the findings. (RAR)
