# Supplementary figures and images for: Studies of Antibiotic Resistance of Beta-Lactamase Bacteria under Different Nutrition Limitations at the Single-Cell Level
Source: PLoS One. 2015 May 20;10(5):e0127115. doi: 10.1371/journal.pone.0127115 (PMC4439059; doi:10.1371/journal.pone.0127115)

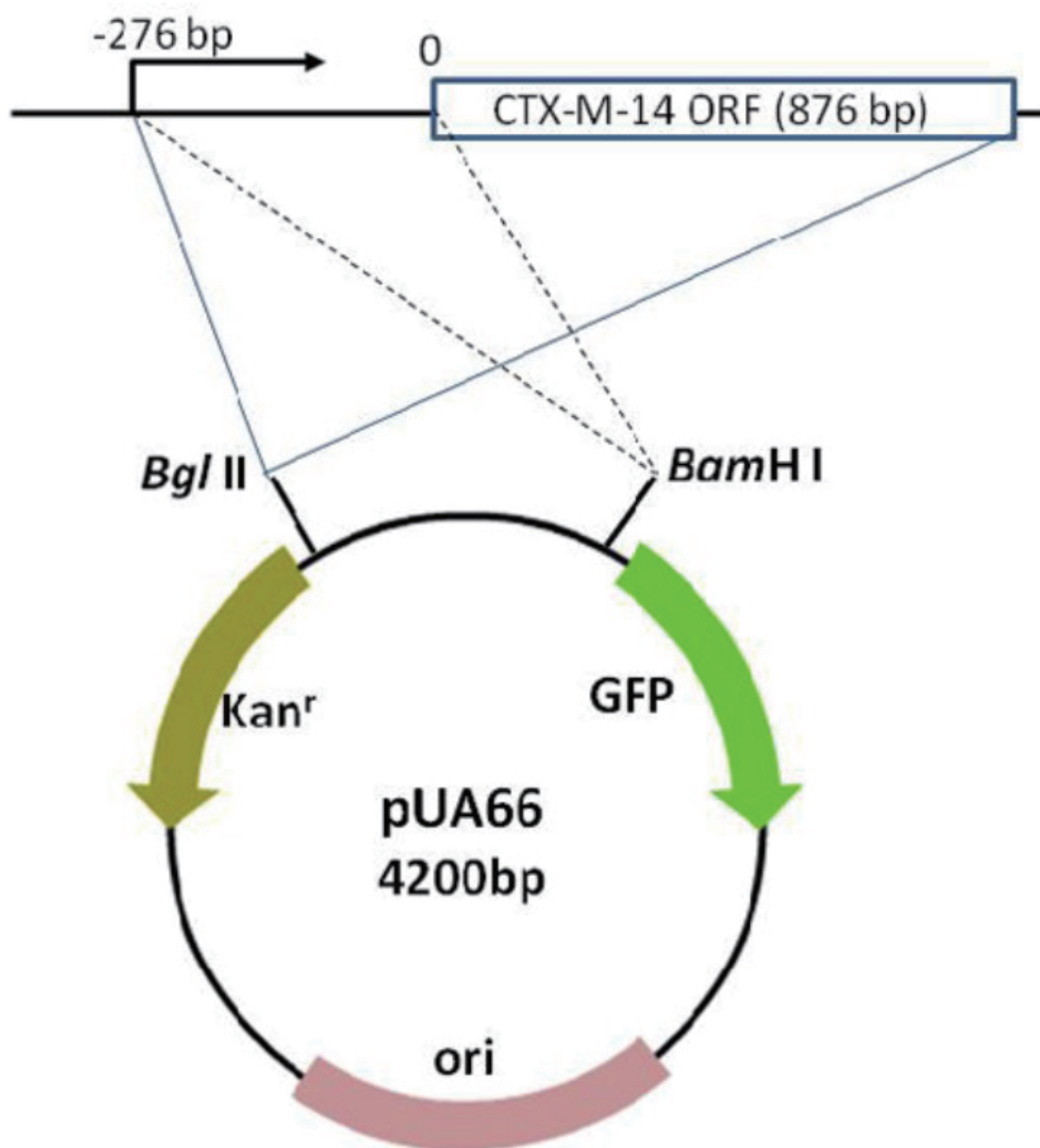

Supplement: S1 Fig — (PDF) [file pone.0127115.s001.pdf]

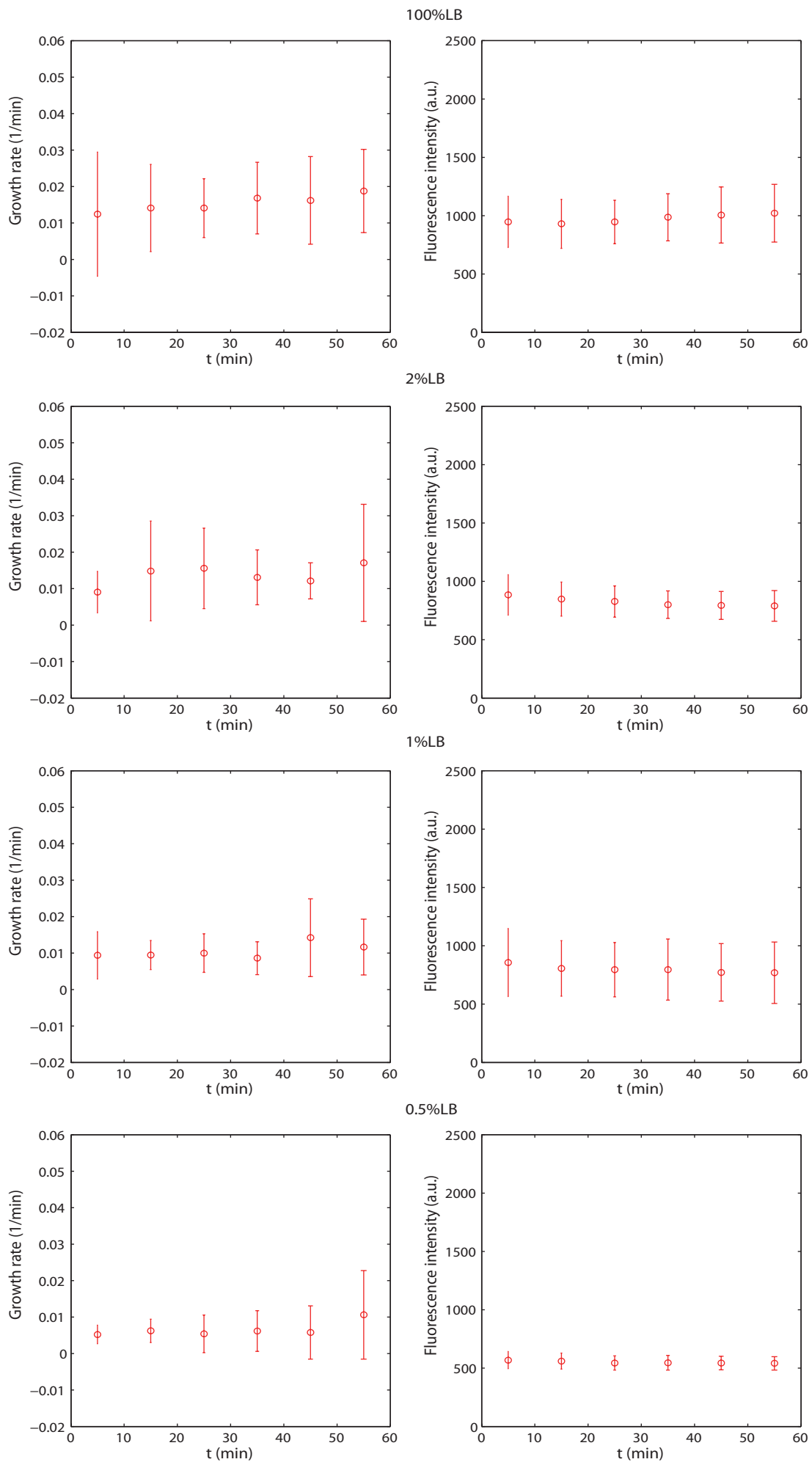

a

b

Supplement: S2 Fig — (PDF) [file pone.0127115.s002.pdf]
