# Supplementary material for: Studies of Antibiotic Resistance of Beta-Lactamase Bacteria under Different Nutrition Limitations at the Single-Cell Level
Source: PLoS One. 2015 May 20;10(5):e0127115. doi: 10.1371/journal.pone.0127115 (PMC4439059; doi:10.1371/journal.pone.0127115)
Supplement: S1 File — (DOC) [file pone.0127115.s003.doc]

**Supporting Information**

**Studies of antibiotic resistance of beta-lactamase bacteria under different nutrition limitations at the single-cell level**

Ying Wang^1^, Min Ran^1^, Jun Wang^2^, Qi Ouyang^1, 2, 3^*, Chunxiong Luo^1, 2^*

^1^The State Key Laboratory for Artificial Microstructures and Mesoscopic Physics, School of Physics, Peking University, Beijing, China

^2^Center for Quantitative Biology, Academy for Advanced Interdisciplinary Studies, Peking University, Beijing, China

^3^Peking-Tsinghua Center for Life Sciences, Peking University, Beijing, China

*To whom correspondence should be addressed: Qi Ouyang, Email: qi@pku.edu.cn; Chunxiong Luo, Email: pkuluocx@pku.edu.cn.

**The medium condition in the chamber is almost identical to that in the main channel**

The chamber’s dimension is 90μm*90μm*1.2μm (9720$\text{μm}^{3}$). However, the flow rate in the main channel is 50μl/hour (50μl/hour=$1.38\times10^{7}\text{μm}^{3}\text{/s}$), which is very fast to renew the medium. Thus the concentration of nutrition and antibiotics are almost constant in the main channel.

The exchange rate between the main channel and the chamber is very fast. It only takes 16 seconds ($\text{L}^{2}/D=(90\text{μm})^{2}/(500\text{μm}^{2}\text{/s})=16.2\text{s}$) to reach equilibrium. Take that the double time for e.coli is 30-60 minutes into consideration, the nutrition consumption rate is very small. Thus, the nutrition and antibiotics condition in the chamber keep equal to the medium in the main channel.

**Strain information**


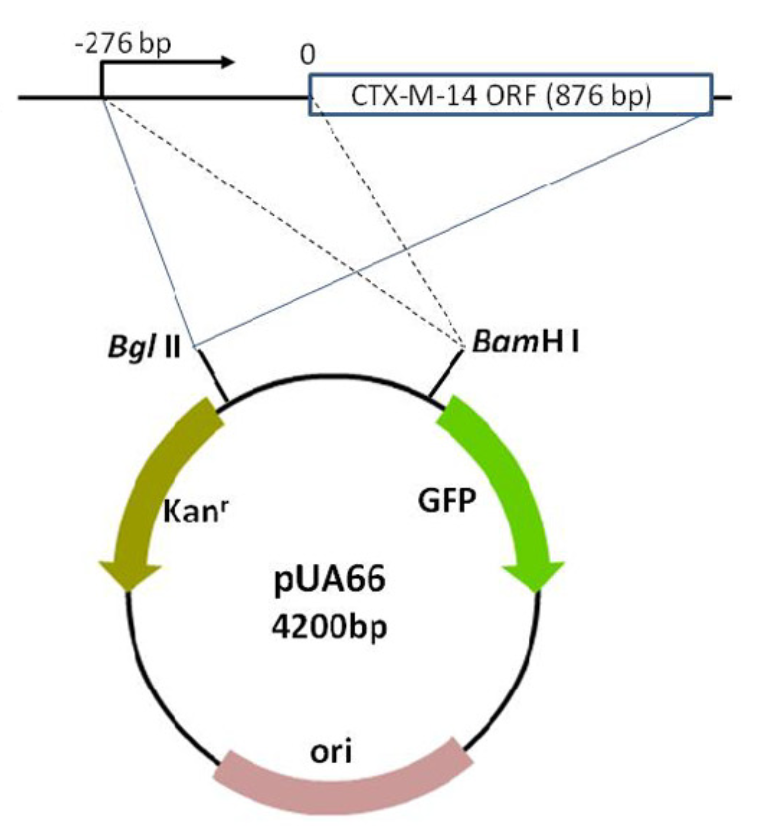


**S1 Fig. Construction of the plasmid expressing CTX-M-14.**

This pUA66 plasmid containing a gene (CTX-M-14) encoding an extended-spectrum beta-lactamase (ESBL, a beta-lactam hydrolase) was imported into an *Escherichia coli* strain, DH5α. We also inserted the CTX-M-14 gene promoter in the upstream region of the green fluorescence protein (GFP) open reading frame (ORF), so that the GFP fluorescence intensity directly reported the expression of the CTX-M-14 gene. We chose the CTX-M-14 gene for the study for its widely known prevalence and the fact that there are no trans regulator or cis elements regulating its expression when antibiotics are functioning.

**Growth rate and fluorescence intensity in the first hour**

**
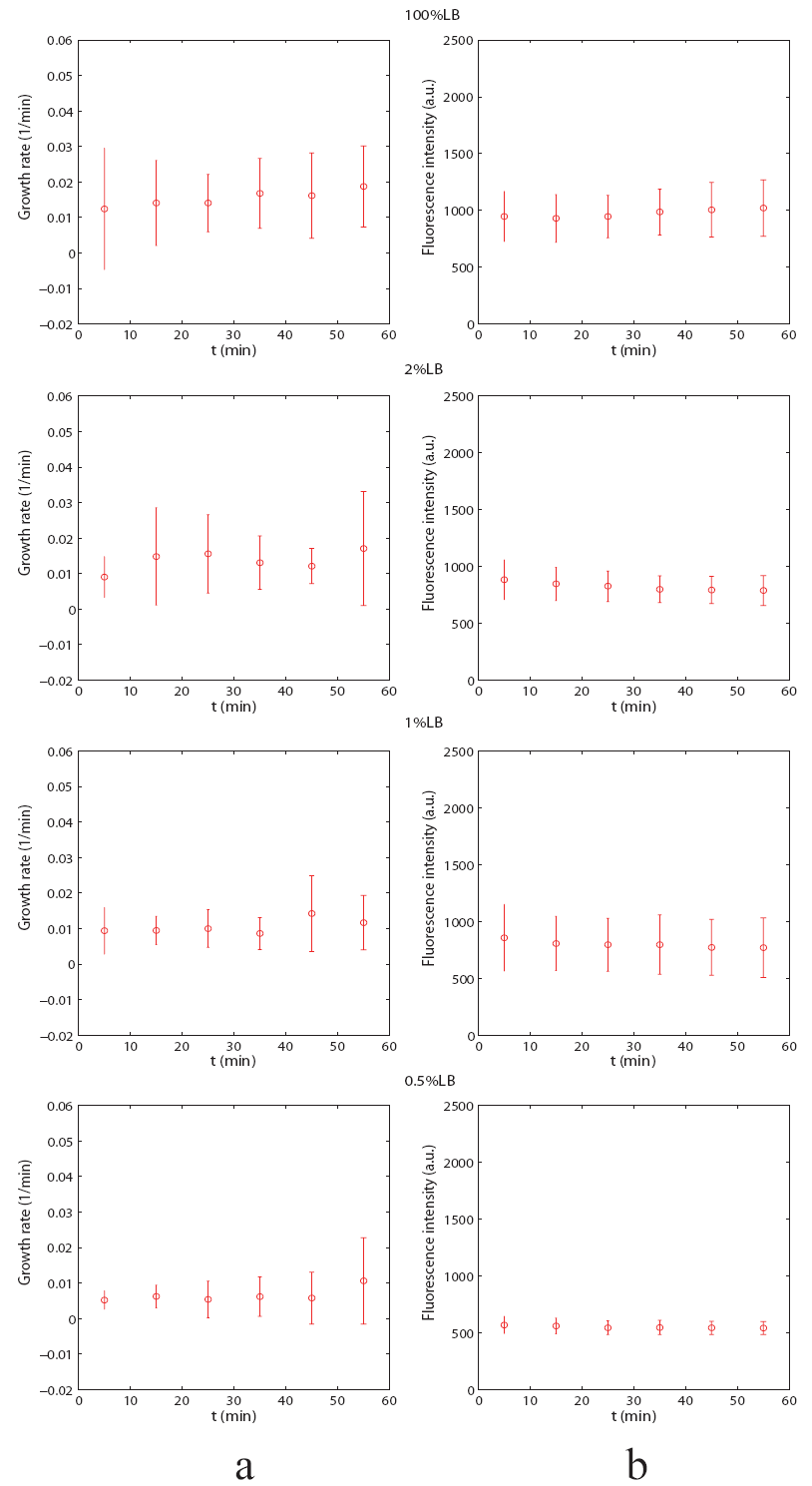
**

**S2 Fig. Growth rate and fluorescence intensity remain constant in the first hour.** (a) The bacterial growth rate was calculated and averaged every 10 minutes in the first hour. The error bar represent the standard deviation of the growth rate. (b) The GFP fluorescence intensity was averaged every 10minutes in the first hour. The error bars represent the standard deviation of the GFP fluorescence intensity.

**References**

[1] A. Zaslaver, A. Bren, M. Ronen, S. Itzkovitz, I. Kikoin, S. Shavit*, et al.*, "A comprehensive library of fluorescent transcriptional reporters for Escherichia coli," *Nat Methods,* vol. 3, pp. 623-8, Aug 2006.

[2] K. Bush and J. F. Fisher, "Epidemiological expansion, structural studies, and clinical challenges of new beta-lactamases from gram-negative bacteria," *Annu Rev Microbiol,* vol. 65, pp. 455-78, 2011.

[3] I. Morrissey, M. Hackel, R. Badal, S. Bouchillon, S. Hawser, and D. Biedenbach, "A Review of Ten Years of the Study for Monitoring Antimicrobial Resistance Trends (SMART) from 2002 to 2011," *Pharmaceuticals (Basel),* vol. 6, pp. 1335-46, 2013.

[4] C. J. C. Wang X R, Kang Y, et al., "Prevalence and characterization of plasmid-mediated blaESBL with their genetic environment in Escherichia coli and Klebsiella pneumoniae in patients with pneumonia," *Chin Med J (Engl),* vol. 125, pp. 894-900, 2012.
